# Mechanism of Solid-State ^1^H Photochemically Induced Dynamic Nuclear Polarization in a Synthetic Donor–Chromophore–Acceptor at 0.3 T

**DOI:** 10.1021/acs.jpclett.4c02805

**Published:** 2024-10-29

**Authors:** Marcel Levien, Federico De Biasi, Ganesan Karthikeyan, Gilles Casano, Máté Visegrádi, Olivier Ouari, Lyndon Emsley

**Affiliations:** ‡ Institut des Sciences et Ingenierie Chimiques, École Polytechnique Fedérale de Lausanne (EPFL), CH-1015 Lausanne, Switzerland; § Aix-Marseille Université, Centre National de la Recherche Scientifique (CNRS), Institut de Chimie Radicalaire, 13013 Marseille, France

## Abstract

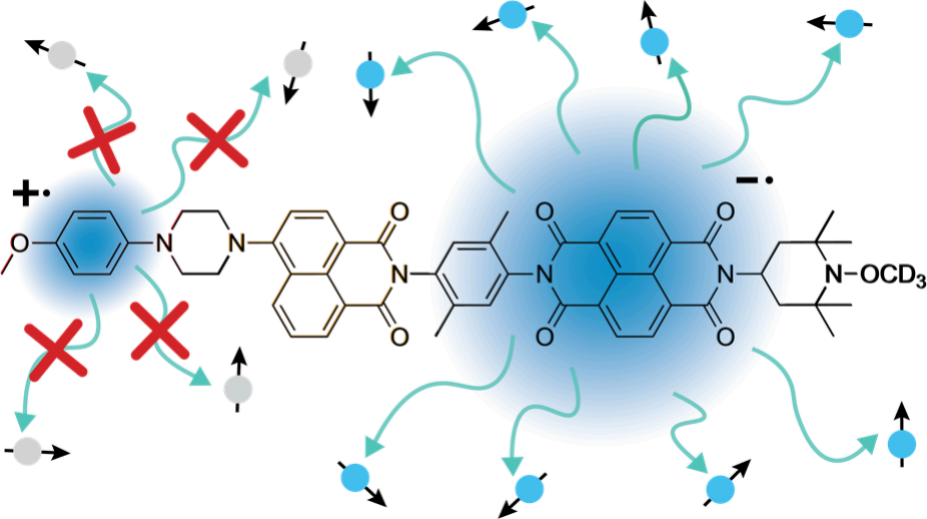

^1^H photochemically induced dynamic nuclear polarization (photo-CIDNP) has recently emerged as a tool to enhance bulk ^1^H nuclear magnetic resonance (NMR) signals in solids at magnetic fields ranging from 0.3 to 21.1 T, using synthetic donor–chromophore–acceptor (D–C–A) molecules as optically active polarizing agents (PAs). However, the mechanisms at play for the generation of spin polarization in these systems have not been determined but are essential for an in-depth understanding and further development of the process. Here, we introduce site-selective deuteration to identify the ^1^H photo-CIDNP mechanisms at 85 K and 0.3 T in D–C–A molecule PhotoPol. We find that the protons on the acceptor moiety are essential for the generation of polarization, establishing differential relaxation as the main mechanism. These results establish selective deuteration as a tool to identify and suppress polarization transfer mechanisms, which opens up pathways for further optimization of the optical PA at both low and high magnetic fields.

Solid-state nuclear magnetic resonance (NMR) is an indispensable tool for elucidating the structure and dynamics of complex systems with applications ranging from physics and materials to chemistry and biology.^1^ Still, as of today, many applications are prevented by the inherently low sensitivity of NMR, stemming from the small energy difference between the nuclear spin states probed in the experiments. The issue is so pressing that by now a whole research area is devoted to dealing with different pathways to alleviate the low sensitivity of NMR by increasing the nuclear spin population imbalance beyond thermal equilibrium, to create hyperpolarization.^2^

Today, most practical applications of hyperpolarization involve microwave-driven dynamic nuclear polarization (DNP) that can lead to significant sensitivity gains. DNP is widely applicable due to the large library of polarization agents (PAs) available today that are compatible with a broad range of sample formulations. However, DNP signal enhancements are limited by the thermal electron spin polarization and cannot exceed a factor of ∼660 for ^1^H spins (the ratio between the gyromagnetic ratios of an electron spin and a ^1^H nuclear spin). This limitation is circumvented if hyperpolarization is achieved by optical methods, as optically excited states can possess much larger out-of-equilibrium spin polarization,^3−6^ in principle, up to unity. Notable examples of such methods are the triplet DNP experiments involving pentacene as the optically active PA.^4,7,8^

Another optical hyperpolarization approach is photochemically induced DNP (photo-CIDNP),^9−14^ which can be active in both liquids and solids. In solid-state photo-CIDNP, hyperpolarization is generated upon optical excitation of a covalently linked donor–acceptor system and subsequent charge separation to form a transient spin-correlated radical pair (SCRP).^15^ Coherent spin evolution between the electronic singlet and triplet states of the SCRP, possibly paired with asymmetries in the nuclear spin relaxation among the singlet and triplet SCRP pathways in the photocycle, can lead to observable nuclear hyperpolarization.^16−18^ Photo-CIDNP in solids was first reported on ^15^N and ^13^C nuclei in photochemical reaction centers and flavoproteins.^19,20^ In the ambit of solid-state photo-CIDNP, most subsequent efforts have been focused on these biological systems, where the technique has served to probe the electronic structure and local environment by directly observing ^13^C and ^15^N nuclear spins.^19−29^ In contrast, only two studies discussed ^1^H photo-CIDNP in biomolecules in solutions, via a solid-state mechanism,^30,31^ or for cyclohexanone in a frozen solution,^32^ via a liquid-state mechanism. In another example ^13^C hyperpolarization was transferred to nearby ^1^H nuclei via cross-polarization.^33^

Recently, we reported bulk ^1^H and ^13^C hyperpolarization in solids via photo-CIDNP using synthetic donor–chromophore–acceptor (D–C–A) molecules at magnetic fields ranging from 0.3 to 21.1 T.^34−36^ Notably, in the case of bulk ^1^H spins, we reported signal enhancements up to a factor of ∼100 at both 9.4 and 21.1 T, demonstrating that such D–C–A systems have the potential to be used in dye-sensitized solid-state NMR studies.^34^ Furthermore, the simple molecular structures of the D–C–A systems are well-suited to rational strategies for optimization of the relevant spin interactions through chemical modification of the PA to obtain more efficient hyperpolarization in the future. Consequently, a deeper understanding of the photo-CIDNP mechanisms and polarization transfer pathways in these systems is required to facilitate the design of this new class of PAs. We note that high-field photo-CIDNP enhanced NMR is likely to be important for many high-resolution magic angle spinning (MAS) studies of modern materials,^1^ while low-field photo-CIDNP could in the future be used as a source of polarization in dissolution DNP-type experiments, which are currently of very high interest in strategies for metabolic imaging.^37^

Here, we identify the mechanism of ^1^H photo-CIDNP in D–C–A systems at 0.3 T. To do this, we employ site-selective deuteration of the photoactive D–C–A system, which is expected to be a powerful probe, as some mechanisms will be suppressed, while others will remain active.^14,38−40^ Following our initial study on bulk ^1^H optical hyperpolarization at 0.3 T using PhotoPol (Figure 1a) as the D–C–A polarizing agent, here, we present two isotopologues where selective deuteration has been applied to the donor or acceptor moiety (panels b and c of Figure 1) to rationalize the polarization transfer mechanism. Upon deuteration of the acceptor, we observe a sharp drop in signal enhancement, clearly showing that differential relaxation (DR) is the dominant photo-CIDNP mechanism in PhotoPol at 0.3 T. More importantly, this proves that selective deuteration is a facile tool to potentially suppress competing polarization transfer pathways and to reveal critical structural motifs on the PA.

**Figure 1:**
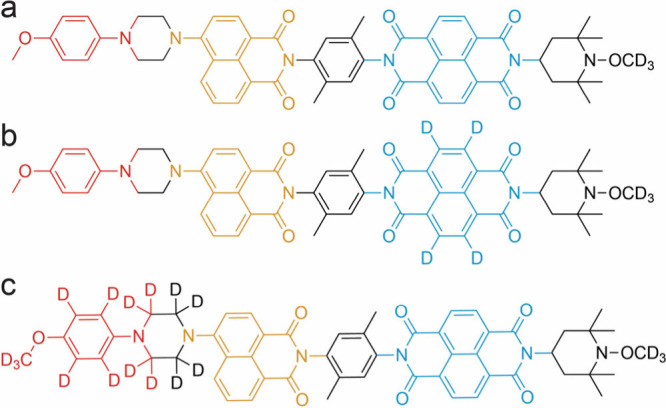
Structure of (a) PhotoPol and its isotopologue with a (b) deuterated acceptor moiety PhotoPol-*d*_A_ and (c) deuterated donor moiety PhotoPol-*d*_D_. The donor 4-methoxyaniline (MeOAn), chromophore 4-aminonaphthalene-1,8-dicarboximide (ANI), and acceptor naphthalene-1,8:4,5-bis(dicarboximide) (NDI) parts of the molecules are highlighted in red, yellow, and blue, respectively. Linkers and end groups are depicted in black.

Solid-state photo-CIDNP can develop nuclear hyperpolarization according to three currently known mechanisms, namely, differential relaxation (DR),^41^ differential decay (DD),^42^ and three-spin mixing (TSM).^18,43^ The smallest spin system needed to describe the process comprises two electron spins and one nuclear spin (*I* = 1/2). In the high magnetic field limit, the truncated Hamiltonian that characterizes the photo-CIDNP process in such a three-spin system can be written as^16^
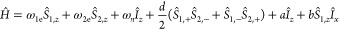
Here, ω_*i*_ is the Larmor frequency of the three spins, and *d* = −2*J*_ex_ – *D* is the electron–electron coupling (with *J*_ex_ and *D* being the exchange and dipolar contributions to the coupling), while *a* and *b* are the secular and pseudo-secular parts of the hyperfine interaction.

Figure 2 shows the typical photocycle of a D–C–A system, such as PhotoPol.^44^ After optical excitation of the chromophore ground state to an excited D–C*–A state, fast (nano- to picosecond) charge separation generates a spin-correlated radical pair consisting of a cationic radical center on the donor site and an anionic radical center on the acceptor site. In PhotoPol and other similar systems, the radical pair is born in the singlet state.^44^ Because the singlet state is not an eigenstate of the high-field spin Hamiltonian, the SCRP undergoes coherent evolution between the |*S*⟩ and |*T*_0_⟩ states, indicated respectively as ^1^(D^•^ ^+^–C–A^•^ ^–^) and ^3^(D^•^ ^+^–C–A^•^ ^–^) in Figure 2. Charge recombination can happen in both the singlet and triplet SCRP states to repopulate the ground state or a neutral triplet state, respectively. Eventually, the neutral triplet also decays to the ground state with a characteristic rate constant. In the case of PhotoPol, the neutral triplet is located on the acceptor,^45^ which is indicated as D–C–^3^A in Figure 2.

**Figure 2:**
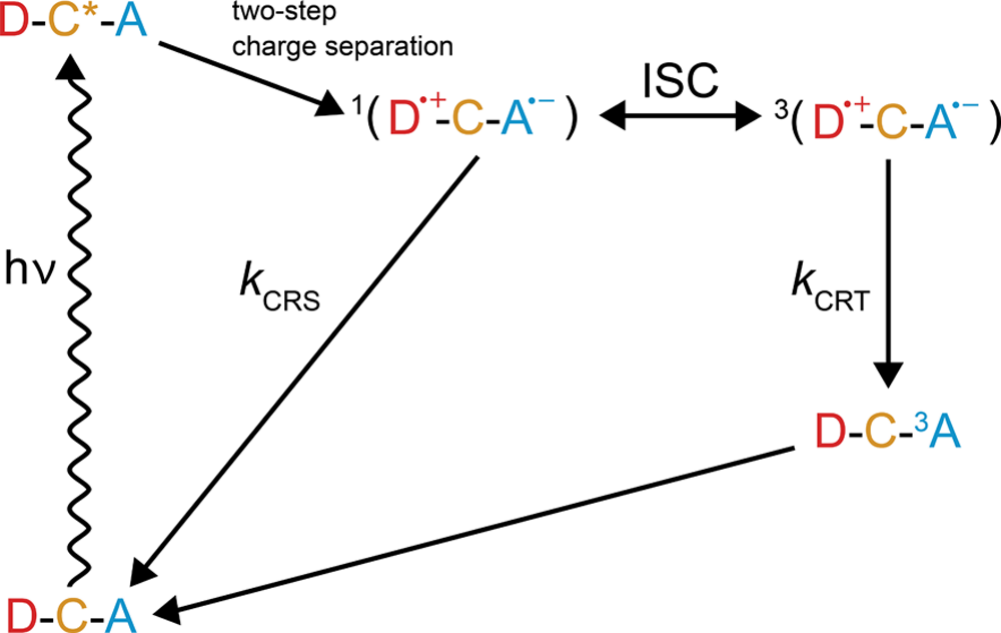
Photocycle in a D–C–A molecule. Photoexcitation by *h*ν is followed by charge separation (ps–ns). Intersystem-crossing (ISC) occurs between the charged singlet and triplet states, which recombine with *k*_CRS_ and *k*_CRT_ rate constants to the ground state and a neutral triplet state, respectively. As indicated, the initial SCRP is formed in the singlet state and the neutral triplet is localized on the acceptor part of the molecule.^44,45^

In solid-state photo-CIDNP, both DR and DD rely on the initial process of nuclear spin sorting among the singlet and triplet SCRP states. Spin sorting occurs because the rate of the |*S*⟩ ↔ |*T*_0_⟩ interconversion depends, among other factors, upon the nuclear spin state and leads to the accumulation of, e.g., |α_n_⟩ nuclear spins in the ^1^(D^•^ ^+^–C–A^•^ ^–^) state and |β_n_⟩ nuclear spins in the ^3^(D^•^ ^+^–C–A^•^ ^–^) state (or vice versa).^46^ In other words, the process of spin sorting causes the accumulation of nuclear spin hyperpolarization in both transient SCRP states with equal magnitude but opposite sign. Spin sorting is most efficient when^16−18^
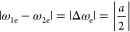
with ω_1e_ being the Larmor frequency of the electron spin coupled to the nuclear spin. However, because of spin sorting, no nuclear spin hyperpolarization is generated in the photocycle, as the nuclear spins have only been distributed among the two SCRP states: at this point, if everything would suddenly decay to the D–C–A ground state, no nuclear spin hyperpolarization would be observed. To convert the sorted nuclear spins into net hyperpolarization, the symmetry between the singlet and triplet channels must be broken.

In differential relaxation, net hyperpolarization is generated due to differences in the nuclear spin longitudinal relaxation time in the singlet and triplet channels. In other terms, the polarization accumulated via spin sorting in one of the two channels is lost because of fast *T*_1,n_ relaxation. Generally, it is expected that *T*_1,n_ is shorter for nuclei that are close to the neutral triplet center, in the case of Figure 2, for nuclei located on the acceptor moiety of the D–C–A structure, because of the paramagnetic relaxation enhancement (PRE) effect. Nonetheless, in the case of PRE, the mechanism is effective only if the neutral triplet lifetime is at least comparable to reduced *T*_1,n_. Notably, DR does not require anisotropic interactions and can be active in the liquid state. The sign of the photo-CIDNP signal enhancement obtained via DR is given by^16,17,47^
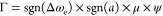
where Δω_e_ = ω_1e_ – ω_2e_, μ = ±1 for a SCRP generated respectively in the triplet or singlet state, and ψ = +1 if the observed polarization is the one generated in the singlet channel and −1 if it is generated in the triplet channel.

In differential decay, nuclear spin hyperpolarization can be generated after spin sorting if the magnitude of the nuclear Larmor frequency is comparable to that of the hyperfine coupling *a*. Whenever this happens, mixing occurs between the |αβα_n_⟩ and |αββ_n_⟩ spin states or, alternatively, between the |βαα_n_⟩ and |βαβ_n_⟩ states, depending upon the sign of *a*. This mixing occurs because the pseudo-secular hyperfine coupling *b* causes coherent nuclear spin flips |α_n_⟩ ↔ |β_n_⟩ in both SCRP singlet and triplet states. These nuclear spin flips deplete with the same rate constant as the polarization accumulates through spin sorting in the singlet and triplet channels. In DD, symmetry between the two channels is broken if the charge recombination rate constants of the singlet and triplet channels (*k*_CRS_ and *k*_CRT_, respectively) differ significantly. Then, the channel with the faster recombination rate constant will be less affected by these nuclear spin flips. For given values of *k*_CRS_ and *k*_CRT_, differential decay is most effective when^16−18,47^
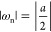
When this resonance condition is met, state mixing is maximized. The sign of the NMR signal enhancement obtained via DD is given by^16,17,47^
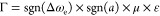
where ε = ±1 for *k*_CRS_ > *k*_CRT_ and *k*_CRS_ < *k*_CRT_, respectively.

Unlike differential relaxation and differential decay, three-spin mixing does not rely on initial nuclear spin sorting between the singlet and triplet manifolds. In TSM, evolution between the SCRP singlet and triplet states involves coherent flip of the coupled nuclear spin (e.g., |*S*α_n_⟩ ↔ |*T*_0_β_n_⟩); therefore, nuclear hyperpolarization is directly generated without the necessity of symmetry breaking. Like DD, TSM requires a pseudo-secular contribution to the hyperfine coupling. In the extreme case of the weak coupling regime (|Δω_e_| ≫ *d*), the matching condition for TSM to occur is^16−18,43^
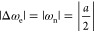
Alternatively, in the strong coupling limit (|Δω_e_| ≪ *d*) the photo-CIDNP effect is maximized when:^16−18,43^
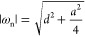
where this resonance condition is derived for |Δω_e_| = 0. In both limiting cases, the sign of the photo-CIDNP enhancement obtained via TSM is determined by^17,43^
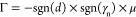
where γ_*n*_ is the gyromagnetic factor of the nucleus. Interestingly, TSM is the only mechanism where the sign of the enhancement is independent of the hyperfine coupling and is expected to be the same for all nuclear spins of the same type (i.e., ^1^H, ^13^C, etc.).

The goal of this study is to rationalize the solid-state photo-CIDNP mechanism in PhotoPol at 0.3 T and 85 K, where we observed a ^1^H bulk enhancement of ε = −16 using *ortho*-terphenyl (OTP) as the glassy matrix.^36^ To this end, it is useful to briefly review the existing spin chemistry data of this system. Figure 3 summarizes isotropic hyperfine coupling constants (HFCs) and their sign as determined from liquid-state electron paramagnetic resonance (EPR) analysis, photo-CIDNP experiments, and density functional theory (DFT) calculations of related compounds.^48−50^ Notably, the reported HFCs were measured in liquids and, therefore, account only for the isotropic contribution of the HFCs *a*_iso_ neglecting the dipolar part. In particular, the pseudo-secular contribution *b* driving DD and TSM is not captured at all in this picture. Determination of the complete hyperfine tensor would be of great value. One avenue could be time-resolved electron paramagnetic resonance (TR-EPR) and time-resolved electron–nuclear double resonance spectroscopy (TR-ENDOR);^51−53^ however, the short lifetime of the SCRP in solids (ns−μs regime)^54^ could potentially be challenging. Additional important parameters on PhotoPol are

- *d*/2π = −5.5 MHz at 85 K^49^
- (ω(NDI) – ω(MeOAn))/2π ≈ −1.2 MHz^49,55^ at 0.3 T
- *k*_CRS_ < *k*_CRT_ in solution at room temperature^49,50,56^
- neutral triplet lifetime in liquid toluene of ∼42 μs^45^
- singlet-born SCRP and neutral triplet localized on the acceptor^44,45^

**Figure 3:**
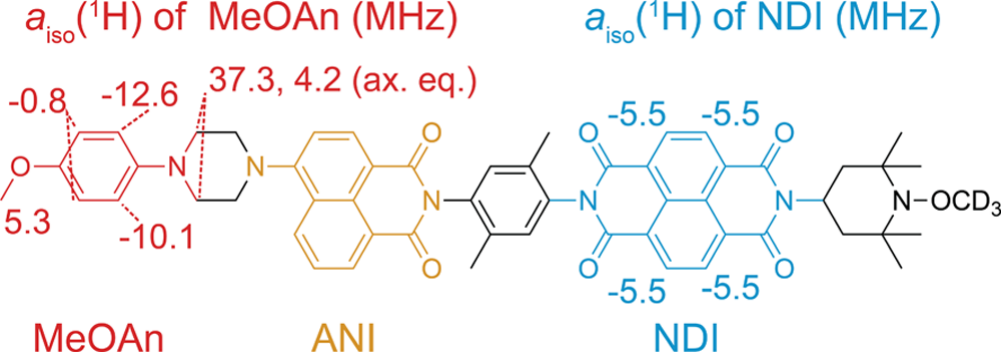
Measured values of the isotropic hyperfine couplings *a*_iso_ in PhotoPol.^48−50^ The donor (MeOAn), chromophore (ANI), and acceptor (NDI) moieties are represented in red, yellow, and blue, respectively. Values reported next to the structure in red and blue are the isotropic hyperfine couplings (MHz) between the ^1^H spins and either the donor (red) or acceptor (blue) radical center, which were previously determined via liquid-state EPR experiments and liquid-state photo-CIDNP experiments of related compounds and where DFT calculations were used to attribute the sign of the couplings.^48−50^ Here, ax and eq refer to axial and equatorial positions with respect to the ring plane.

Figure 4 shows the ^1^H signal enhancement of *ortho*-terphenyl in photo-CIDNP experiments using 1 mM PhotoPol and its selectively deuterated analogues PhotoPol-*d*_A_ and PhotoPol-*d*_D_. Details on the synthesis and the experimental procedure are given in the Experimental Methods and Supplementary Section 1 of the Supporting Information.

**Figure 4:**
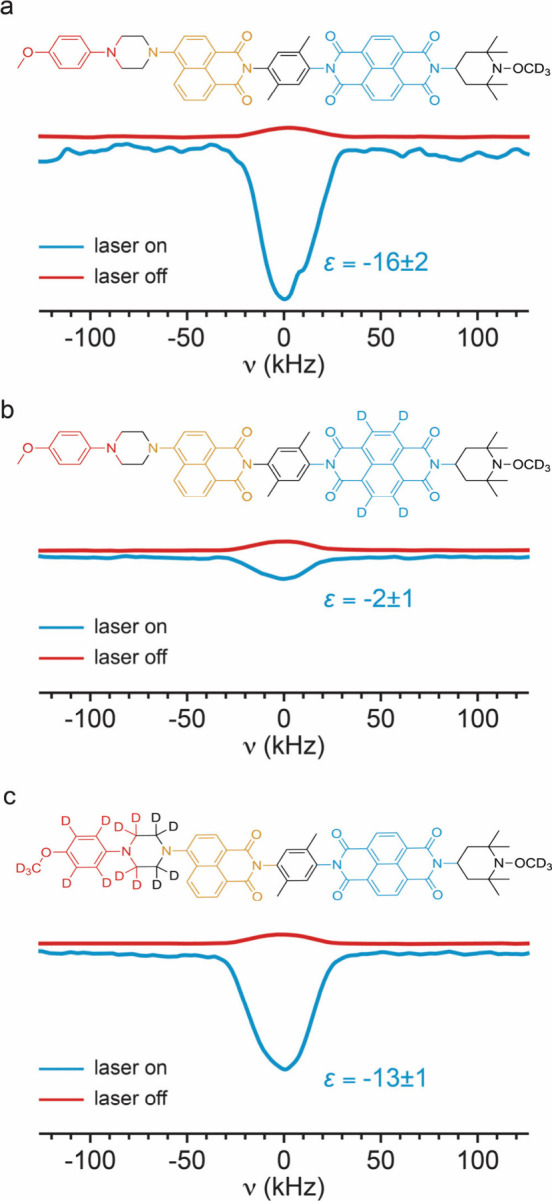
^1^H NMR spectra of OTP at 0.3 T and 85 K using 1 mM (a) PhotoPol, (b) PhotoPol-*d*_A_, and (c) PhotoPol-*d*_D_ as the PA. Blue spectra were acquired using continuous wave (cw) laser illumination (3.8 W/cm^2^) for (a and c) 50 scans or (b) 200 scans, while red spectra were acquired without laser illumination for (a) 10 000 or (b and c) 3000 scans. For better comparison, laser on spectra in panels a–c were normalized to their respective laser off spectrum. Spectra were acquired as an echo (15 μs delay) with a polarization delay of 20 s. More information on the pulse sequence, pre-saturation, and ringing suppression are given in the Experimental Methods. The data in panel a were reported in ref (36).

We observe a steep drop in signal enhancement from ε ≈ −16 to −2 upon deuteration of the protons located on the acceptor part (NDI) of the molecule (panels a and b of Figure 4). On the other hand, as seen in Figure 4c, almost no change in signal enhancement is observed if the protons on the donor part (MeOAn) of the molecule are deuterated (ε ≈ −13).

As observed, the four NDI protons are crucial for the photo-CIDNP mechanism. If *k*_CRS_ < *k*_CRT_ and the signs of the liquid-state HFCs also hold at 85 K, the sign rules for the signal enhancement exclude DD as the dominant mechanism because that would predict a positive enhancement for the acceptor and, therefore, suggest TSM or DR as the main polarization mechanism for PhotoPol at 85 K. However, as stated earlier, using only liquid-state HFC data would not allow for a reliable conclusion on the origin of photo-CIDNP hyperpolarization. Instead, we note that the neutral triplet state is localized on the NDI moiety,^45^ which suggests DR as the main polarization mechanism. This is supported by the fact that deuteration of the donor has an almost negligible influence on the enhancement. If TSM was the main cause of hyperpolarization, we would expect a larger change in enhancement upon deuteration of the donor part of PhotoPol, because all ^1^H spins in the D–C–A system sharing a HFC with the SCRP should be active for this mechanism.

We note in the case of PhotoPol-*d*_A_ a reduced *T*_1,n_ (Figure S2 of the Supporting Information), which is attributed to the partial degradation of the PA (Figure S14 of the Supporting Information). To exclude any influence of the buildup time and *T*_1,n_ on the observed enhancements, we also report in Figure S3 of the Supporting Information the steady-state signal enhancements measured with a polarization delay of ∼5*T*_1,n_. Apart from slightly smaller overall enhancements, the results confirm the trend reported in Figure 4.

In conclusion, we have reported the synthesis of two selectively deuterated PhotoPol analogues and the corresponding solid-state ^1^H photo-CIDNP bulk signal enhancements in OTP at 0.3 T and 85 K. The obtained signal enhancement, taken together with spin chemistry data from the literature,^44,45,48−50,54−57^ shows that DR is the dominant polarization mechanism for PhotoPol at 0.3 T and 85 K. This demonstrates how selective deuteration can be a fruitful tool to identify and potentially suppress polarization transfer pathways in solid-state photo-CIDNP. This could potentially be used to maximize ^1^H hyperpolarization at low and high magnetic fields. Future studies will involve experiments at high magnetic fields to investigate the ^1^H photo-CIDNP polarization mechanisms at magnetic field strengths relevant for high-resolution NMR under MAS.

## Experimental Methods

*Synthesis of Deuterated PhotoPol Derivatives and Other Materials*. Details on the synthesis of the deuterated compounds are provided in the Supporting Information. The synthesis of PhotoPol was already reported.^36^ All of the other compounds were used as received.

*Sample Preparation*. Appropriate amounts of the two deuterated PhotoPol derivatives were dissolved in toluene to yield stock solutions with a concentration of ∼1 mM PA. About 10 μL of the stock solution was transferred to a 3 mm (outer diameter) NMR glass tube, and toluene was evaporated. Afterward, ∼11 mg of *ortho*-terphenyl was added to the tube. The sample was then melted [mp(OTP) = 65 °C] under a vacuum using a hot water bath. Several freeze–pump–thaw cycles were performed under a vacuum to remove dissolved oxygen. For each cycle, the sample was melted in a water bath. To increase the degassing efficiency, OTP was allowed to crystallize in between cycles. Cycles were repeated until no more bubbles during melting were observed (usually ∼3 cycles).

*NMR Setup*. A Varian electromagnet was used to generate a magnetic field of 0.3 T. The saddle NMR coil was shielded in a copper cavity. Radio-frequency (rf) pulse generation and signal acquisition was performed using a PulseBlaster SpinCore pulse generator, a PTS 620 frequency synthesizer, a TOMCO rf pulse amplifier, a GaGe-applied RazorMax digitizer, and a home-built spectrometer. Tuning and matching capacitors (NMTIMI20CEK) are placed in an aluminum box next to the cryostat. The copper cavity is placed in a Bruker ER 4118CF cryostat, which is used to stabilize the sample temperature at 85 K (using liquid N_2_). Copper cavities and cryostats have an optical window that allows for light irradiation of the sample. Irradiation was performed using a 450 nm continuous wave laser coupled to a λ/2 waveplate and a polarizing beam splitter to adjust the output power. The light reaches the optical window through an optical fiber connected to a collimator. The diameter of the beam is about 3 mm, corresponding to a 7 mm^2^ irradiation area. Power levels given in this publication were measured at the end of the optical fiber using a thermal power sensor and, considering the losses across the different optical windows, are expected to be reduced at the sample position.

*NMR Pulse Sequences*. Pulse sequences for all experiments are reported in Figure S1 of the Supporting Information. The rf frequency was centered at ∼12.76 MHz (^1^H resonance frequency). Acoustic ringing was suppressed using a 180° inversion pulse before the solid echo on even scans and the inversion of the receiver phase. The 90° and 180° pulse lengths were 3.5 and 7 μs, respectively. Spectra were acquired using the following parameters: τ_sat_ = 0.1 ms, τ_rs_ = 0.5 μs, and τ_SE_ = 15 μs. Spectra in Figure 4 and Figure S3 of the Supporting Information were acquired with τ_rec_ = 20 and 200 s, respectively. For buildup and *T*_1,n_ measurements (Figure S2 of the Supporting Information), τ_rec_ was varied between 1 and 500 s. In total, 20 equally spaced hard 90° pulses were used for pre-saturation. All data were background-subtracted and phased, and laser on spectra were normalized to the intensity of the respective laser off spectrum. Data in Figure 4a were reported in Figure 3 of ref (36).

## Supporting Information Available

Synthetic methods and characterization of deuterated PhotoPol derivatives, supplementary figures and tables, and a link to all of the raw NMR data (PDF)
