# Supplementary material for: Mechanism of Solid-State 1H Photochemically Induced Dynamic Nuclear Polarization in a Synthetic Donor–Chromophore–Acceptor at 0.3 T
Source: J Phys Chem Lett. 2024 Oct 29;15(44):11097–103. doi: 10.1021/acs.jpclett.4c02805 (PMC11552079; doi:10.1021/acs.jpclett.4c02805)
Supplement: Supplementary file 1 — jz4c02805_si_001.pdf [file jz4c02805_si_001.pdf]

Mechanism of solid-state ^{1}H photo-CIDNP in a synthetic donor-chromophore-acceptor at 0.3 T

Marcel Levien,[1+] Federico De Biasi,[1+] Ganesan Karthikeyan,[2] Gilles Casano,[2] Máté Visegrádi,[1] Olivier Ouari,[2*] and Lyndon Emsley[1,*]

[1] *Institut des Sciences et Ingenierie Chimiques, École Polytechnique Fedérale de Lausanne (EPFL), CH-1015 Lausanne, Switzerland*

[2] *Aix-Marseille Univ, CNRS, Institut de Chimie Radicalaire, 13013 Marseille, France*

Raw NMR data

All the raw NMR data associated with the manuscript can be accessed at the following link DOI: 10.5281/zenodo.13991089 and is available under the CC-BY-4.0 (Creative Commons Attribution-ShareAlike 4.0 International) license.

Summary

1) Pulse sequence....................S1

2) Buildup and T_{1n} experiments....................S2

3) Steady-state enhancements....................S3

4) Synthetic procedures....................S4

5) References....................S4

1) Pulse sequence:

The experimental setup and pulse sequence were optimized in a previous study[1] and were adopted here. Figure S1 displays the pulse sequence used in all reported experiments. For buildup and T_{1n} studies the delay t_{rec} was incremented between 1 s and 200 s (500 s for PhotoPol-d_D). The additional 180° pulse on even scans together with a shift of the receiver phase suppresses acoustic ringing significantly preventing severe baseline distortions.

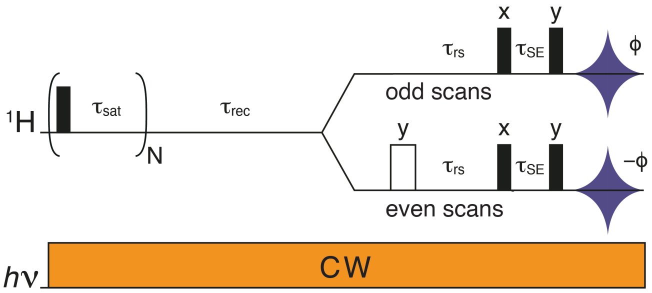

Figure S1: Pulse sequence used for all NMR experiments reported in the present study. Full rectangles represent 90° pulses, while open rectangles are 180° pulse. Details on the acquisition parameters are given in the Methods section. A two-step phase cycle enables suppression of acoustic ringing. Figure from ref.1.

2) Buildup and T_{1n} measurements:

Buildup and T_{1n} were recorded for PhotoPol-d_A as well as for PhotoPol-d_D. We note a reduced T_{1n} of PhotoPol-d_A that we attribute to some sample degradation. Experimental data were fitted to an exponential function of the form $I(t) = A \cdot (1 - \exp\left(-\frac{\tau}{T_i}\right))$, where T_i is either the buildup time T_b or the nuclear relaxation time T_{1n}. All data were background subtracted. To reduce sample degradation during the acquisition of the buildup curves, these experiments were performed with reduced laser power (P = 2.4 W/cm^2).

Table S1: Number of scans (ns) for each data point in the buildup and saturation recovery curves of Photopol-d_A and PhotoPol-d_D.

t_{rec} (s)	ns laser off, PhotoPol-d_A	ns laser on, PhotoPol-d_A	ns laser off, PhotoPol-d_D	ns laser on, PhotoPol-d_D
1	60000	6000	56000	1000
2	12000	2500	12000	600
5	8000	1000	10000	300
10	4000	400	4000	160
20	3000	200	3000	50
50	500	100	500	30
100	400	40	500	16
200	300	30	300	8
500	–	–	155	6

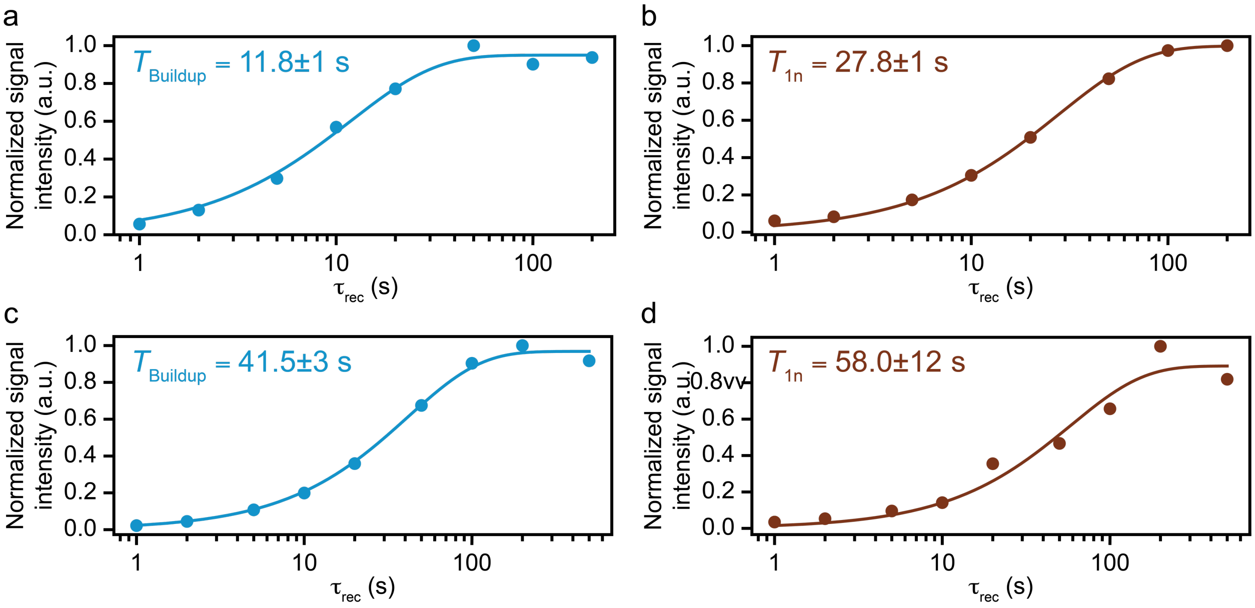

Figure S2: Buildup (a,c) and saturation recovery (b,d) experiments of PhotoPol-d_A (a-b) and PhotoPol-d_D (c-d). Saturation recovery experiments were carried out without laser irradiation, while for buildup experiments continuous laser irradiation was applied. Solid lines are monoexponentially fits to $I(t) = A \cdot (1 - \exp\left(-\frac{\tau}{T_i}\right))$ where T_i is either the buildup time T_b or the nuclear relaxation time T_{1n} yielding the values reported in the inset.

3) Steady-state enhancements:

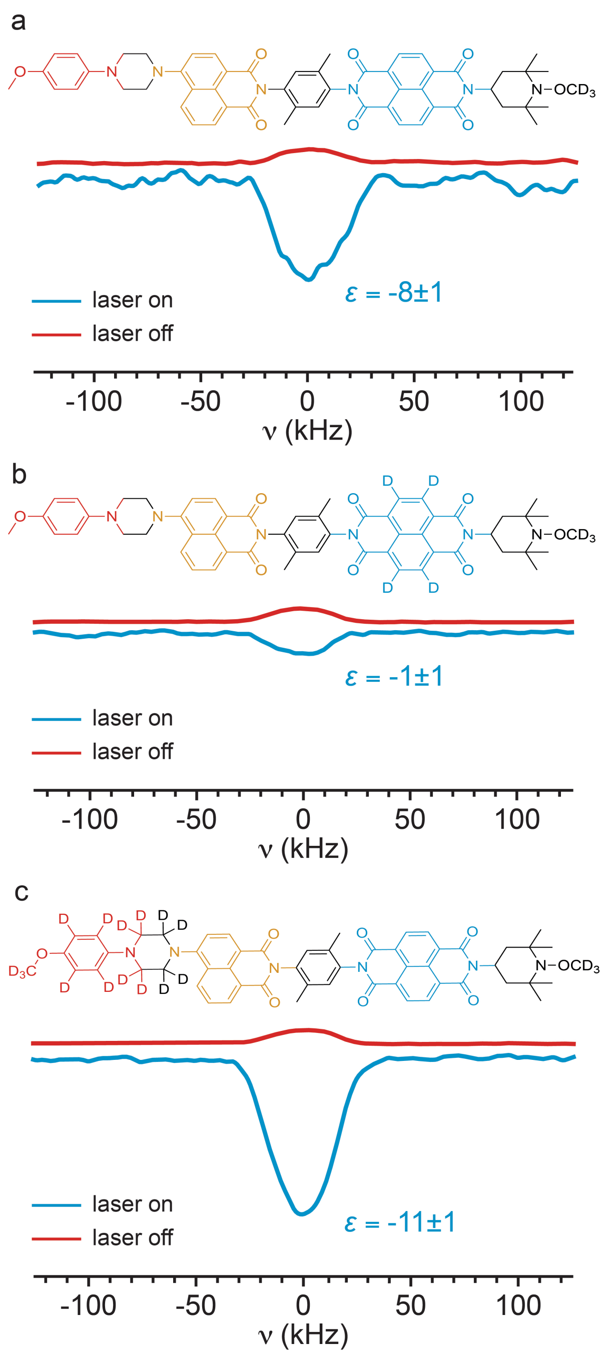

Figure S3: ^{1}H NMR spectrum of OTP at 0.3 Tesla and 85 K using 1 mM of (a) PhotoPol, (b) PhotoPol-d_A, and (c) PhotoPol-d_D as the PA. Blue spectra were acquired using cw laser illumination (3.8 W/cm^2) for 8 scans, while red spectra were acquired without laser illumination for 300 scans (a) and 200 scans (b and c). For better comparison, spectra in a-c were normalized to their respective laser off spectrum. Spectra were acquired as an echo (15 µs delay) with a polarization delay of 200 s. More information on the pulse sequence, pre-saturation, and ringing suppression are given in the methods. (a) was already reported Figure S4 of ref.[1].

4) Synthetic procedure:

General procedures. Unless otherwise noted, all reactions were carried out under an inert atmosphere of argon. Chemicals were used as received from the suppliers. Reactions were monitored by thin-layer chromatography (TLC) analysis. Column chromatography was carried out on silica gel (230-400 mesh). The ^{1}H NMR spectra were recorded on a Bruker AVL spectrometer at 300 MHz and 400 MHz. The chemical shifts are reported in ppm downfield relative to TMS and referenced using the residual $CHCl_3$ resonance (δ = 7.26) for ^{1}H NMR. ESI-HRMS were performed on a SYNAPT G2 HDMS (Waters). LCMS was performed on Agilent 1260 Infinity, 6120 Quadrupole. The NMR and MS analysis were performed at the Spectropole facilities, Marseille. The starting material **A**[2] and **B**[3] were prepared using reported procedures starting from commercially available anisole-d_8 and piperazine-d_8, respectively.

Synthesis of PhotoPol-d_D (7)

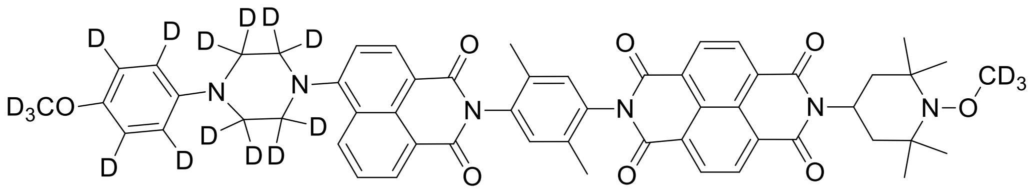

PhotoPol-d_D (7)

Synthesis of starting material 1

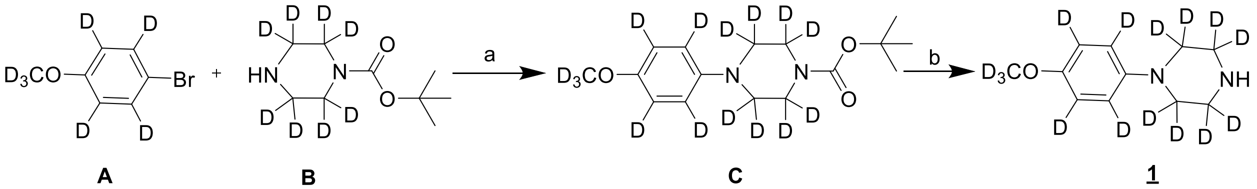

Scheme S1: Reagents and conditions: a) JohnPhos, KO*t*Bu, $Pd_2(dba)_3$, dioxane, 50°C, 5hrs. b) HCl / dioxane, MeOH, 2hrs.

Synthesis of compound C

To the mixture of bromoarene **A** (0.1g, 0.5 mmol) and amine **B** (0.1g, 0.5 mmol) in dioxane (5ml) were added JohnPhos (40 mg, 25 mol %), potassium *tert*-butoxide (0.13g, 1.15 mmol) and tris(dibenzylideneacetone)palladium (30 mg, 5 mol%). Then the reaction mixture was degassed with argon for 15 min and heated at 50°C for 5hrs. The reaction was quenched with aqueous NH_4Cl solution (5 mL) at room

temperature and extracted with dichloromethane (2x20 mL). The organic layer was washed with brine, dried with sodium sulfate and concentrated in vacuo. The crude was purified by column chromatography to give compound **C** (0.1g, 65%), R_f = 0.1 (10% Ethylacetate / Pentane).

HRMS (ESI-TOF) m/z: [M + Na]$^+$ calcd. for $C_{16}H_9D_{15}N_2O_3Na^+$ 330.2621; found 330.2621.

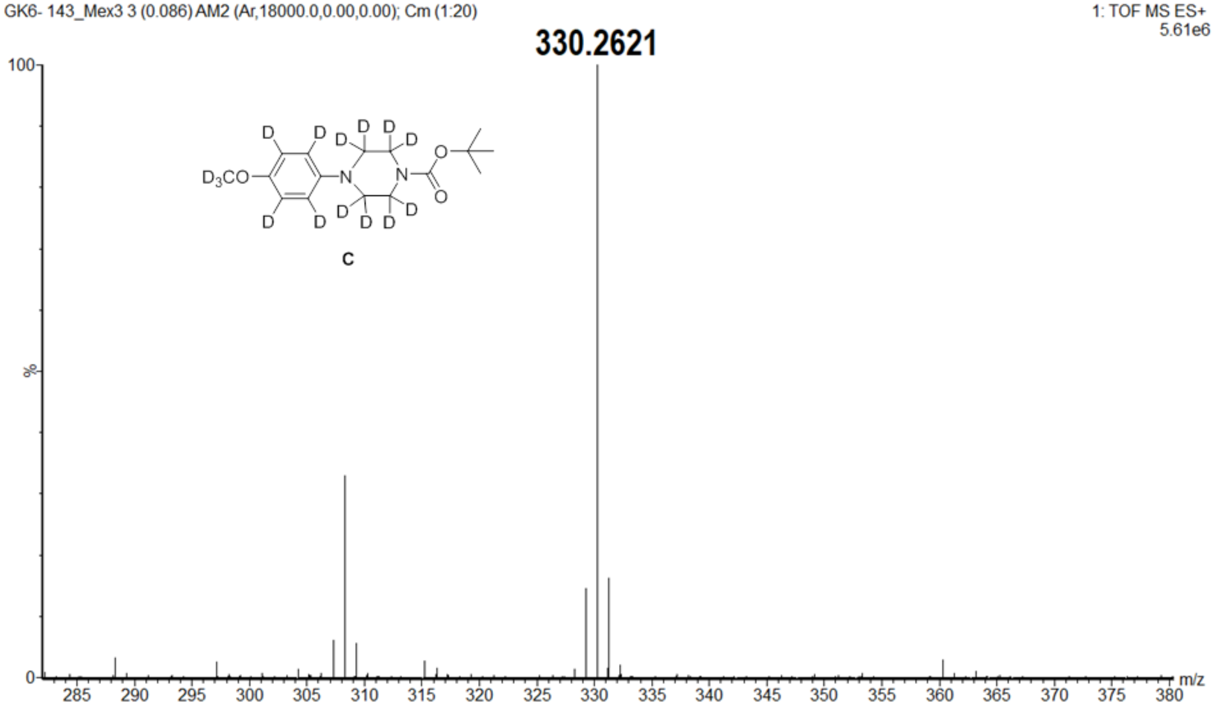

Figure S4: ESI-HRMS spectrum of compound **C**

Synthesis of compound 1

To the solution of compound **C** (90 mg, 0.3 mmol) in methanol was added 4.0 M HCl in dioxane and stirred at RT for 2hrs. The reaction mixture was diluted with water (20 mL) and pH was adjusted to 8 -9 using triethylamine and extracted with dichloromethane (10 ml x 2 times). The combined organic layer was washed with brine and dried with sodium sulfate and concentrated in vacuo to give compound **1** as brown solid in quantitative yield.

LC-MS (ESI) m/z: 208.2 [M+H]$^+$.

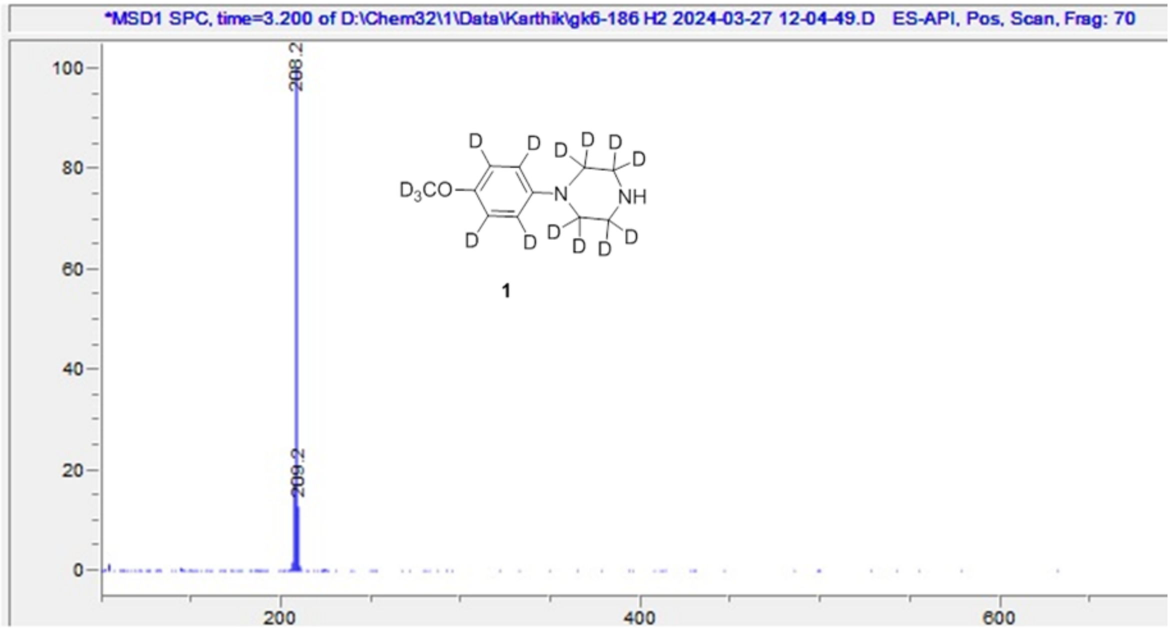

Figure S5: ESI-MS spectrum of compound **1**

Synthetic scheme for PhotoPol-d$_D$ (7)

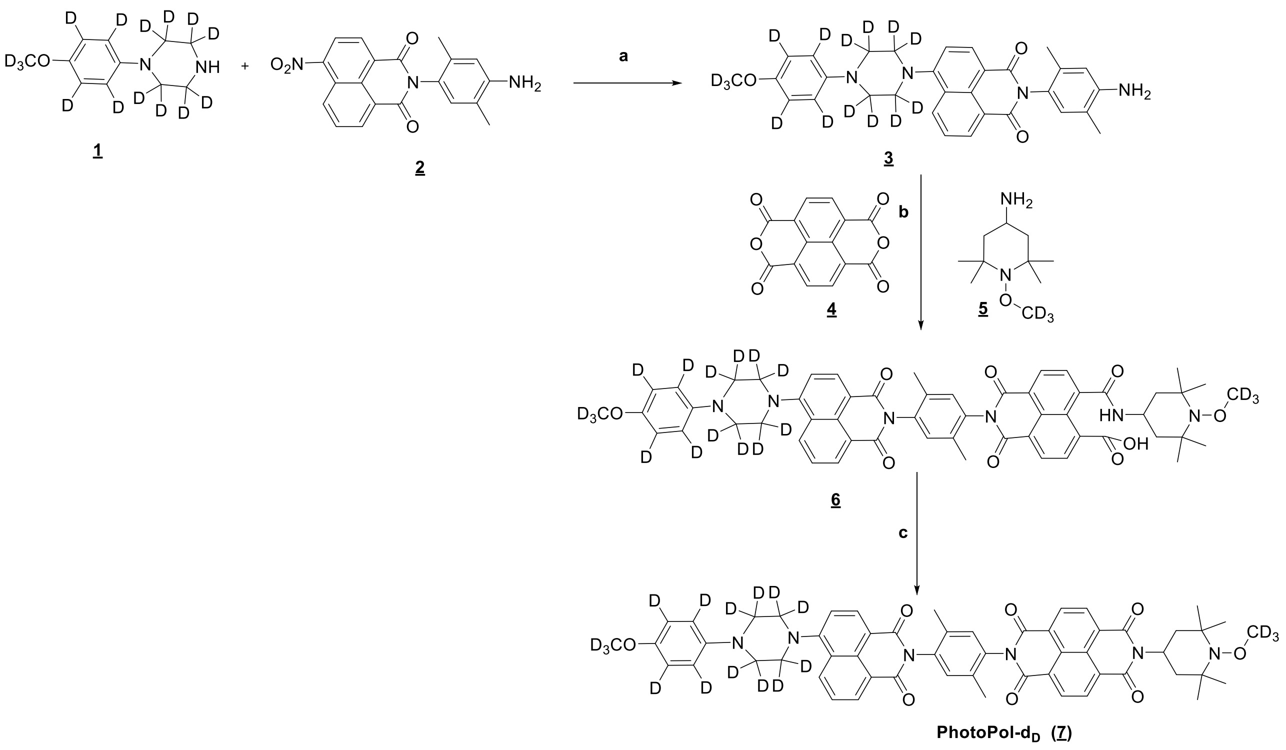

Scheme S2: Reagents and conditions: a) NMP, 120 °C, 2hrs. b) Compounds 4 and 5, Pyridine, 120 °C, 16hrs, c) Ac_2O, $CHCl_3$, 75 °C, 4hrs.

Synthesis of compound 3

A mixture of compound **1** (0.1g, 0.48 mmol) and **2** (0.11g, 0.32 mmol) in *N*- Methyl pyrrolidine (4 ml) was degassed with argon and heated at 120° C for 2 hrs. The reaction mixture was cooled to room temperature and water was added to obtain a precipitated solid. The solid was filtered, dried and purified by column chromatography to give compound **3** (80 mg, 50 %), R_f = 0.34 (10% Acetone/ CH_2Cl_2).

^{1}H NMR ($CDCl_3$, 300 MHz) δ= 2.04 (s, 3H), 2.15 (s, 3H), 6.67 (s, 1H), 6.87 (s, 1H), 7.7-8.0 (m,1H), 8.4-8.8 (m,4H).

HRMS (ESI-TOF) m/z: $[M + H]^+$ calcd for $C_{31}H_{16}D_{15}N_4O_3^+$ 522.3332; found 522.3333.

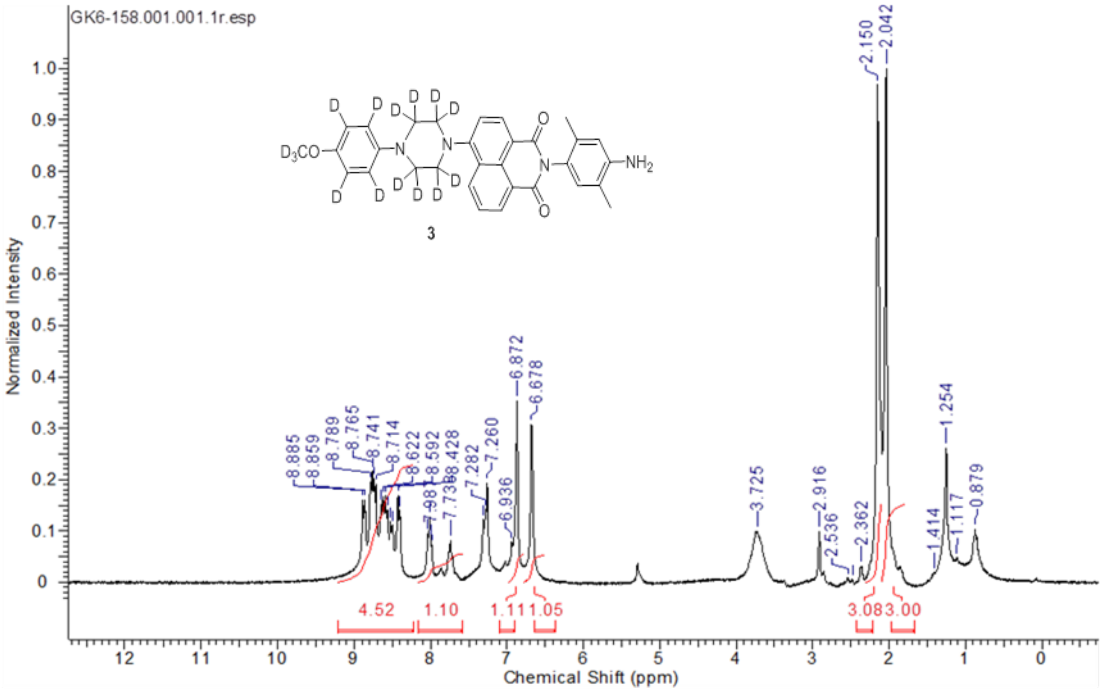

Figure S6: ^{1}H NMR Spectrum of compound **3**.

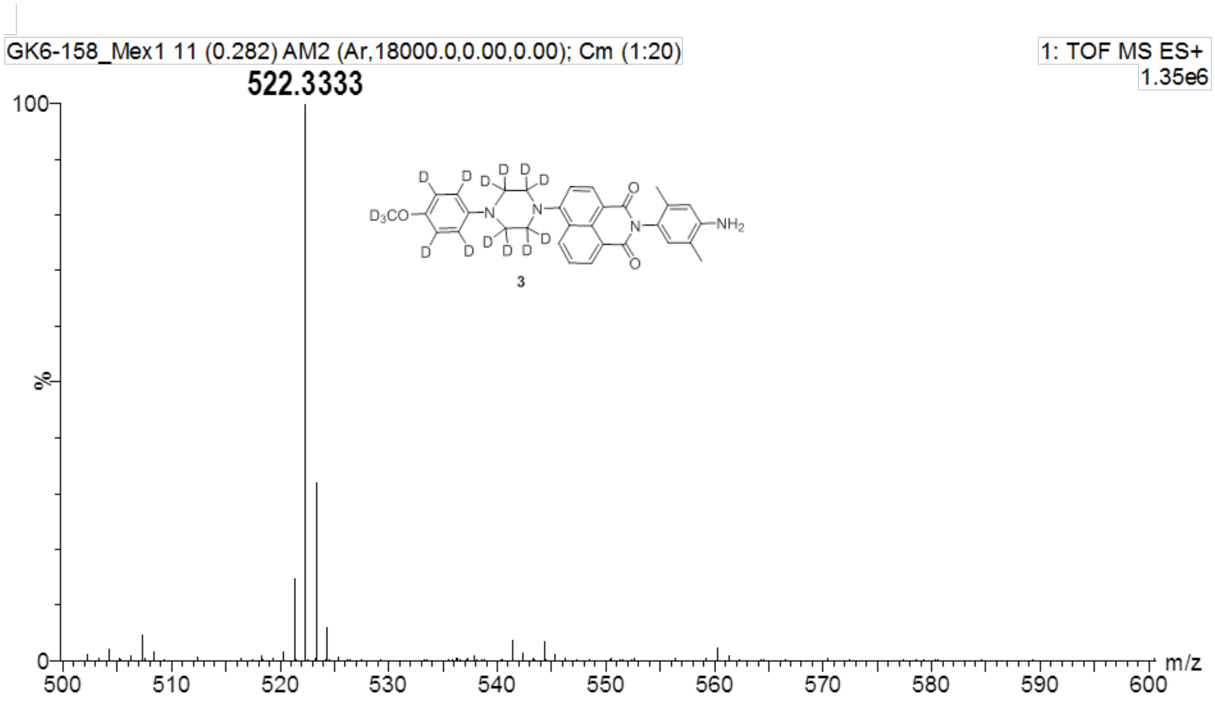

Figure S7: ESI-HRMS spectrum of compound **3**

Synthesis of compound 6

Compound **3** (55 mg, 0.10 mmol) and 1, 4, 5, 8-naphthalenetetracarboxylic dianhydride **4** (28 mg, 0.1 mmol) were dissolved in 3mL of pyridine and the mixture was heated at 110 °C under an argon atmosphere for 5 hours. Then compound **5** (20 mg, 0.1 mmol) in 0.1mL pyridine was added and the reaction mixture was continued heating at 110 °C for 3 hrs. The reaction mixture was concentrated in vacuum and purified by column chromatography to give compound **6** (26mg, 27 %), R_f = 0.3 (10% MeOH/ CH_2Cl_2) and PhotoPol-d_D (**7**,3.7 mg, 4 %), R_f = 0.7 (10% MeOH/ CH_2Cl_2).

LC-MS (ESI) m/z: 961.4 $[M+H]^+$.

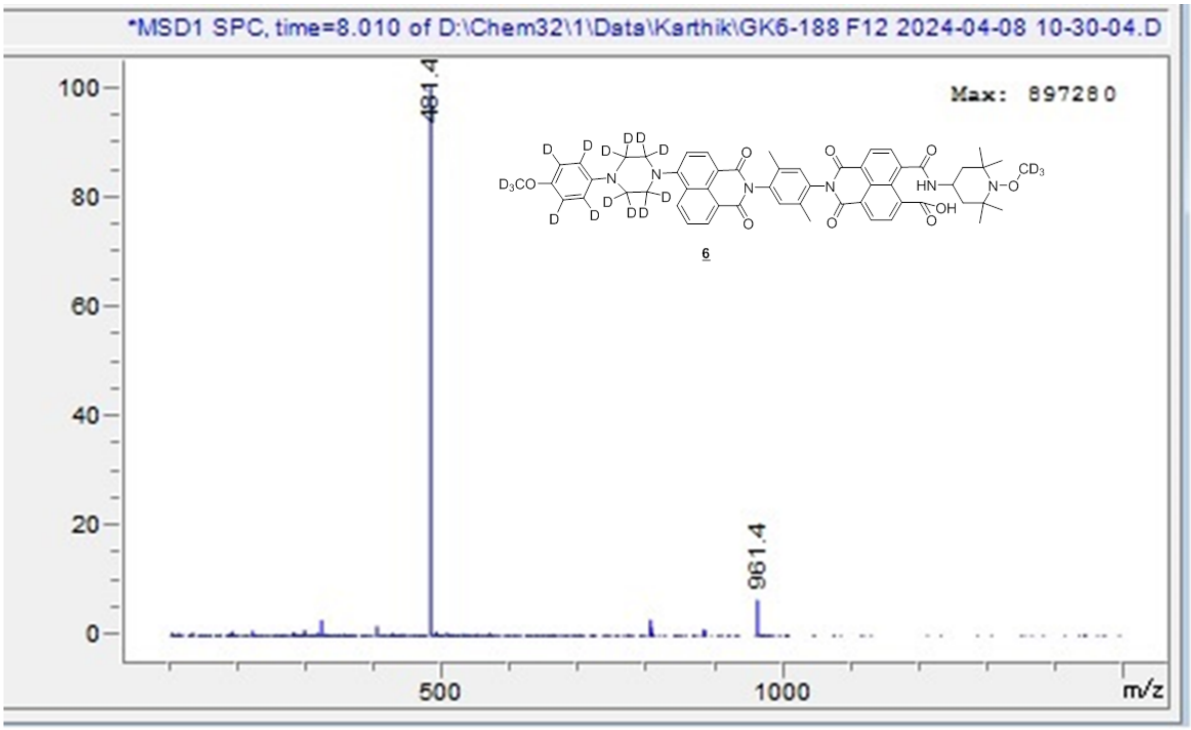

Figure S8: ESI-MS Spectrum of compound **6**

Synthesis of PhotoPol-d_D (**7**)

Compound **6** (13 mg, 0.01 mmol) in $CHCl_3$ was treated with acetic anhydride (50 µL) and heated at 75 °C for 4hrs. The reaction mixture was concentrated in vacuum and purified by preparative thin layer chromatography to give **PhotoPol-d$_D$** (**7**) (6 mg, 47%), R_f = 0.7 (10% MeOH/CH_2Cl_2).

^{1}H NMR ($CDCl_3$, 400 MHz) δ= 1.18-1.26 (m, 12H), 1.70-1.79(m, 4H), 2.11 (s, 3H), 2.13 (s, 3H), 3.66-3.68 (m, 1H), 7.18-7.22 (m,2H), 7.24-7.28 (m,2H), 7.69-7.73 (m,1H), 8.47 (dd, J= 8.4, 1.2 Hz, 1H), 8.55 (d, J= 8.0 Hz, 1H), 8.61 (dd, J= 7.2, 1.2 Hz, 1H), 8.7-8.77 (m, 3H).

HRMS (ESI-TOF) m/z: [M + Na]$^+$ calcd for $C_{55}H_{34}D_{18}N_6O_8Na^+$ 965.4869; found 965.4868.

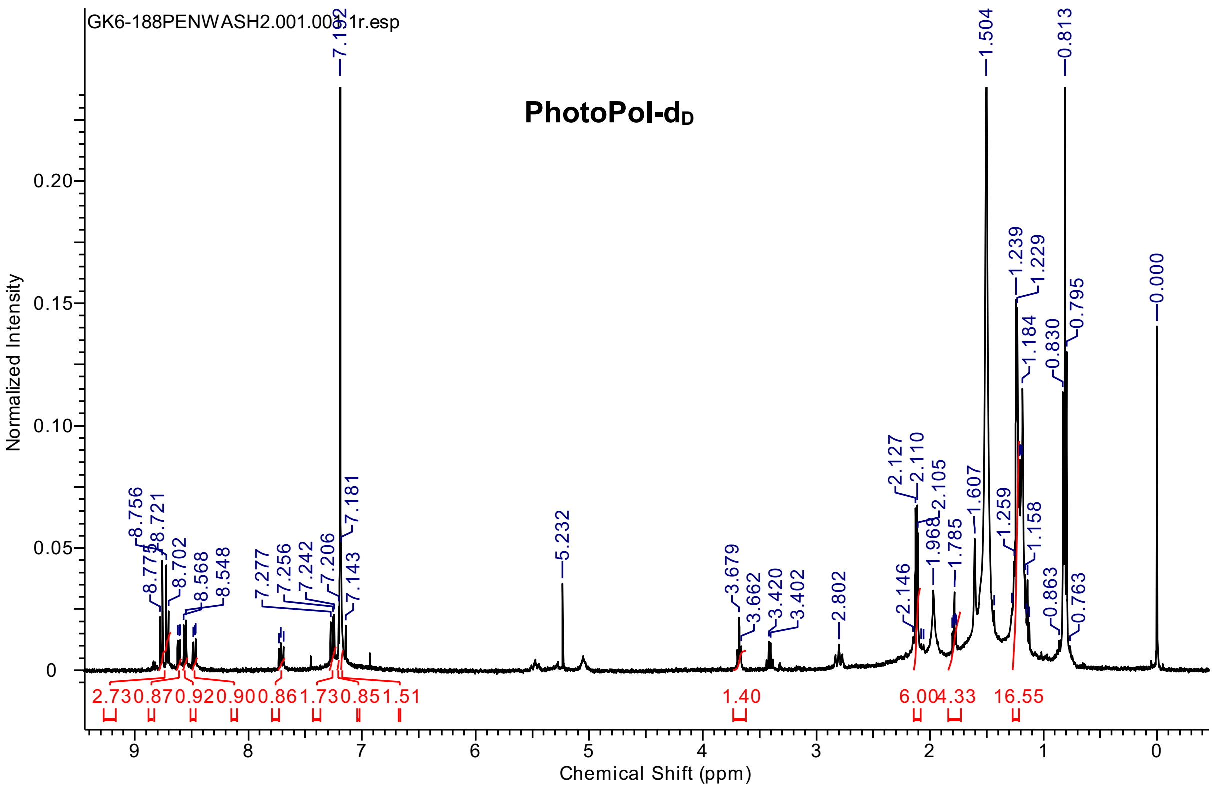

Figure S9: ^{1}H NMR Spectrum of PhotoPol-d$_D$ (7).

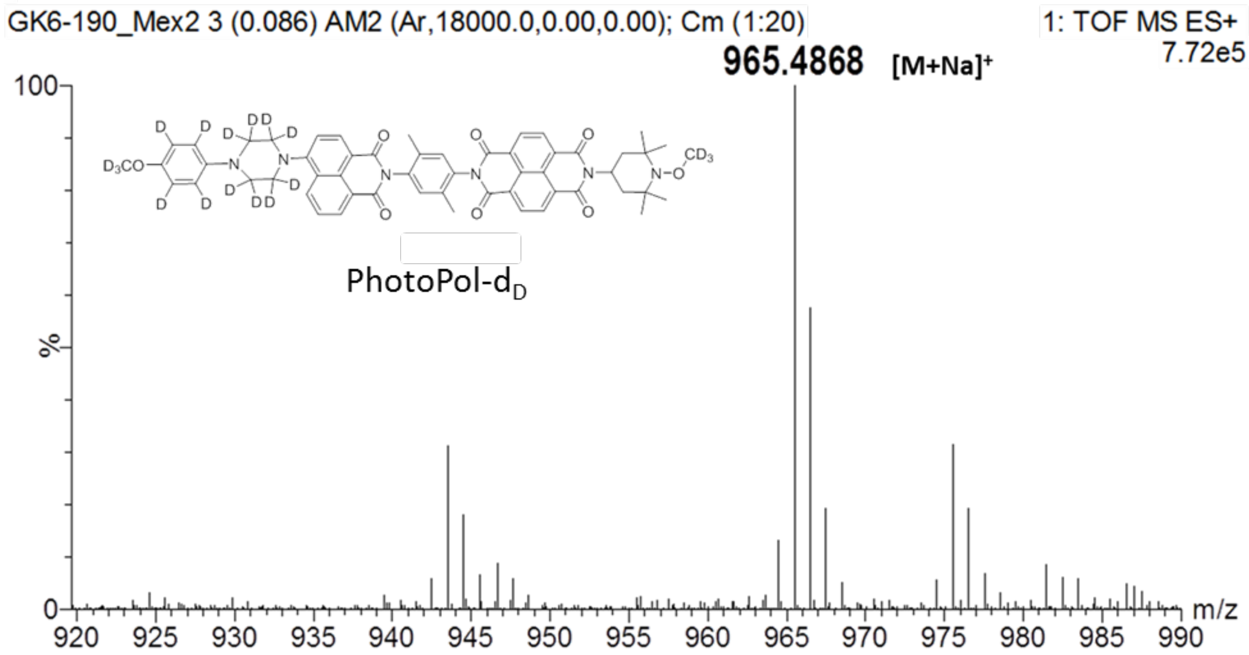

Figure S10: ESI-HRMS spectrum of **PhotoPol-d$_D$** (7)

Synthesis of PhotoPol-d$_A$ (12)

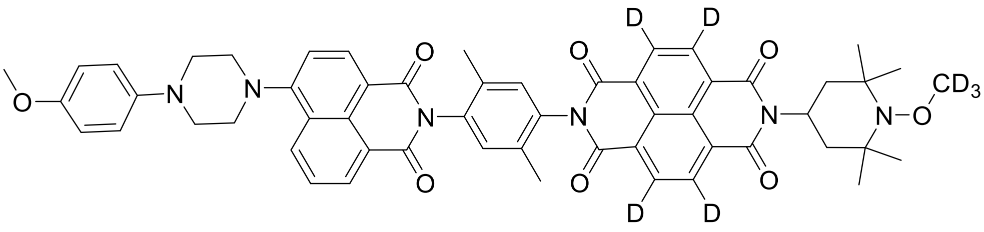

PhotoPol-d$_A$ (12)

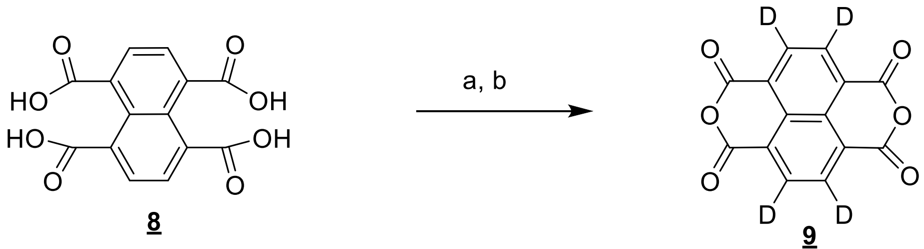

Scheme S3: Synthetic scheme for compound 9: a) Pt/C 5%, D_2O, H_2 (5 bars), 180°C, 12h. b) AcOH, MWI, 120°C, 30 min.

Synthesis of compound 9

1,4,5,8-Naphtalenetetracarboxylic acid **8** (0.75 g, 2.45 mmol) was added in pure D_2O (15 mL). A drop of NaOD (1N) was added to obtain a pH around 7. After stirring 10 minutes under argon atmosphere, the mixture was filtered. Only a part of the 1,4,5,8-naphtalenetetracarboxylic acid was dissolved. The colored filtrate and Pt/C (0.1 g) were placed in a sealed tube under hydrogen atmosphere (5 bars) and the reaction was stirred at 180°C for 12h. After cooling, the mixture was filtrated and the solution was concentrated under reduced pressure to give a brown compound (40 mg). To control the deuterium incorporation, ^{1}H NMR sample was realized in DMSO-d6 with *tert*-butanol as a reference. No aromatic signal was observed. The brown compound (40 mg) was dissolved in acetic acid (3 mL) and the solution was stirred under microwave irradiation (120°C, 30 min.). After cooling, the precipitate was filtered and washed with diethyl ether to obtain compound **9** as solid and was used without further purification.

Synthetic scheme for PhotoPol-d$_A$ (12)

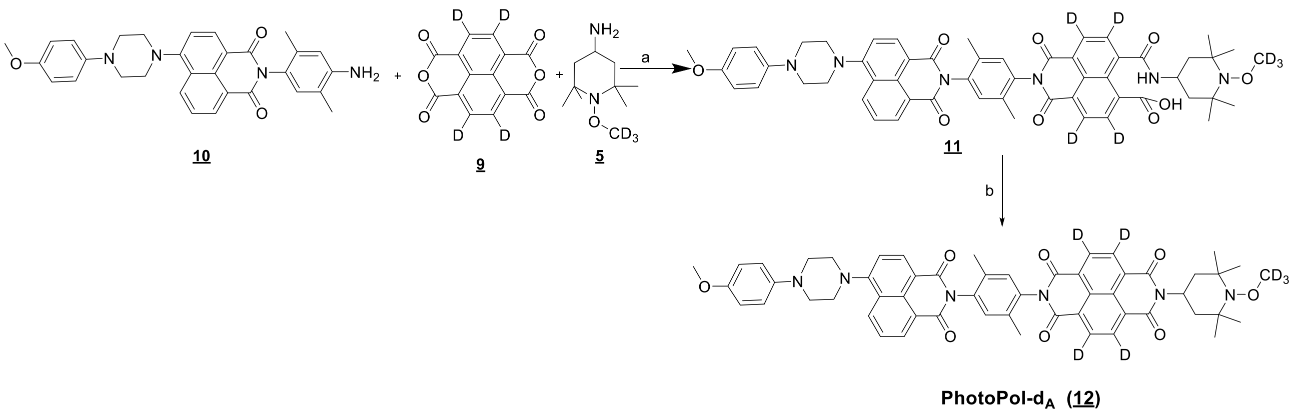

Scheme S4: Reagents and conditions: a) Compound 9, Pyridine, 110 °C, 4 hrs, Compound 5, 110 °C, 16 hrs. b) Ac_2O, $CHCl_3$, 75 °C, 4hrs.

Synthesis of compound 11.

Compound **10** (25 mg, 0.05 mmol) and deuterated naphthalene tetracarboxylic dianhydride **9** (13 mg, 0.05 mmol) were dissolved in 3mL of pyridine and the mixture was heated at 110 °C under an argon atmosphere for 4hours. Then compound **5** (20 mg, 0.1 mmol) in 0.1mL pyridine was added and the reaction mixture was continued heating at 110 °C for 16 hrs. The reaction mixture was concentrated in vacuum and purified by column chromatography to give compound **11** (15mg, 31 %), R_f = 0.28 (10% MeOH/ CH_2Cl_2).

HRMS (ESI-TOF) m/z: 950.4 $[M+H]^+$; 972.4 $[M+Na]^+$

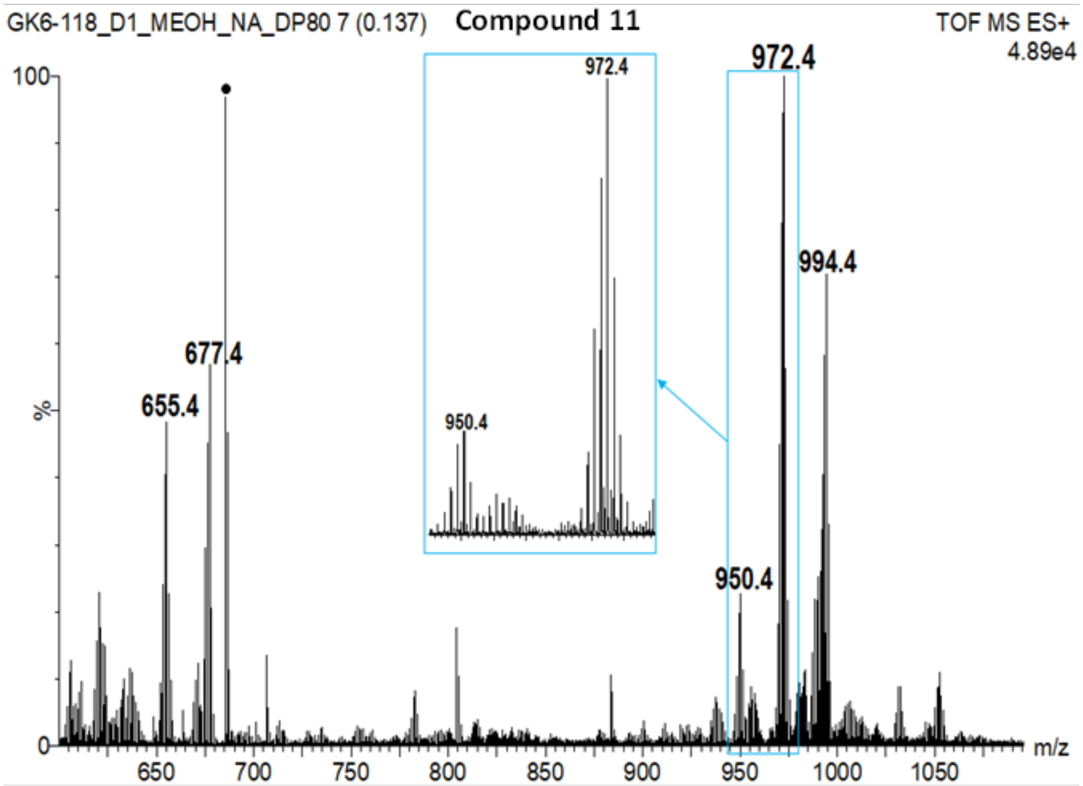

Figure S11: ESI-HRMS spectrum Compound **11**

Synthesis of PhotoPol-d$_A$ (12)

Compound **11** (15 mg, 0.015 mmol) in $CHCl_3$ was treated with acetic anhydride (50 µL) and heated at 75 °C for 4hrs. The reaction mixture was concentrated in vacuum and purified by preparative thin layer chromatography to give PhotoPol-d$_A$ **12** (8 mg, 57%), R_f = 0.71 (10% MeOH/CH_2Cl_2).

^{1}H NMR ($CDCl_3$, 400 MHz) δ= 1.26-1.31 (m, 12H), 1.72-1.85 (m, 4H), 2.17 (s, 6H), 3.43-3.49 (m, 9H), 3.82(s, 3H), 6.90-6.93 (m,2H), 7.02-7.04 (m,2H), 7.27 (d, J= 9.6 Hz, 2H), 7.34 (d, J= 8.0 Hz 1H), 7.76-7.80 (m,1H), 8.55 (dd, J= 8.4, 0.8 Hz, 1H), 8.63 (d, J= 8.0 Hz, 1H), 8.68 (dd, J= 7.2, 0.8 Hz, 1H).

HRMS (ESI-TOF) m/z: 932.4 [M+H]$^+$; 954.4 [M+Na]$^+$

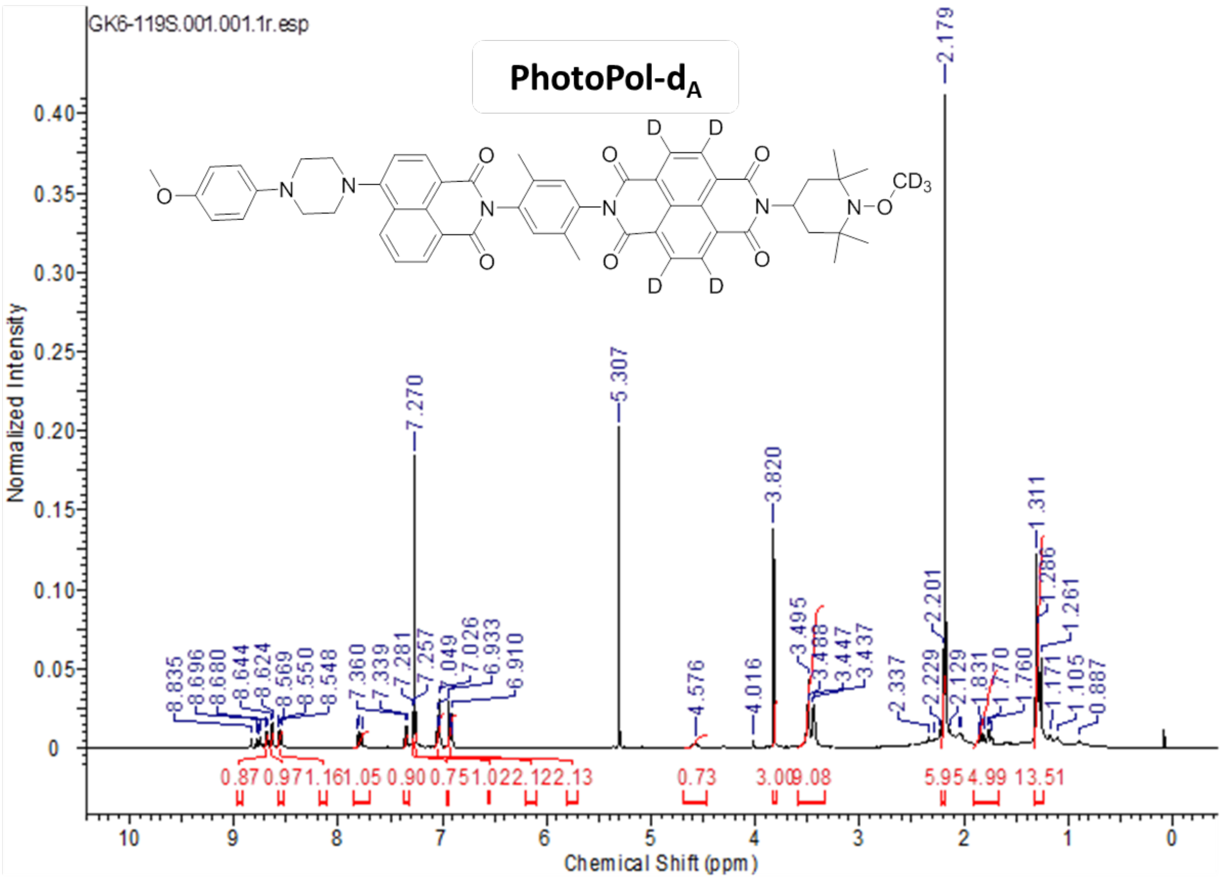

Figure S12: ^{1}H NMR Spectrum of **PhotoPol-d$_A$ (12)**

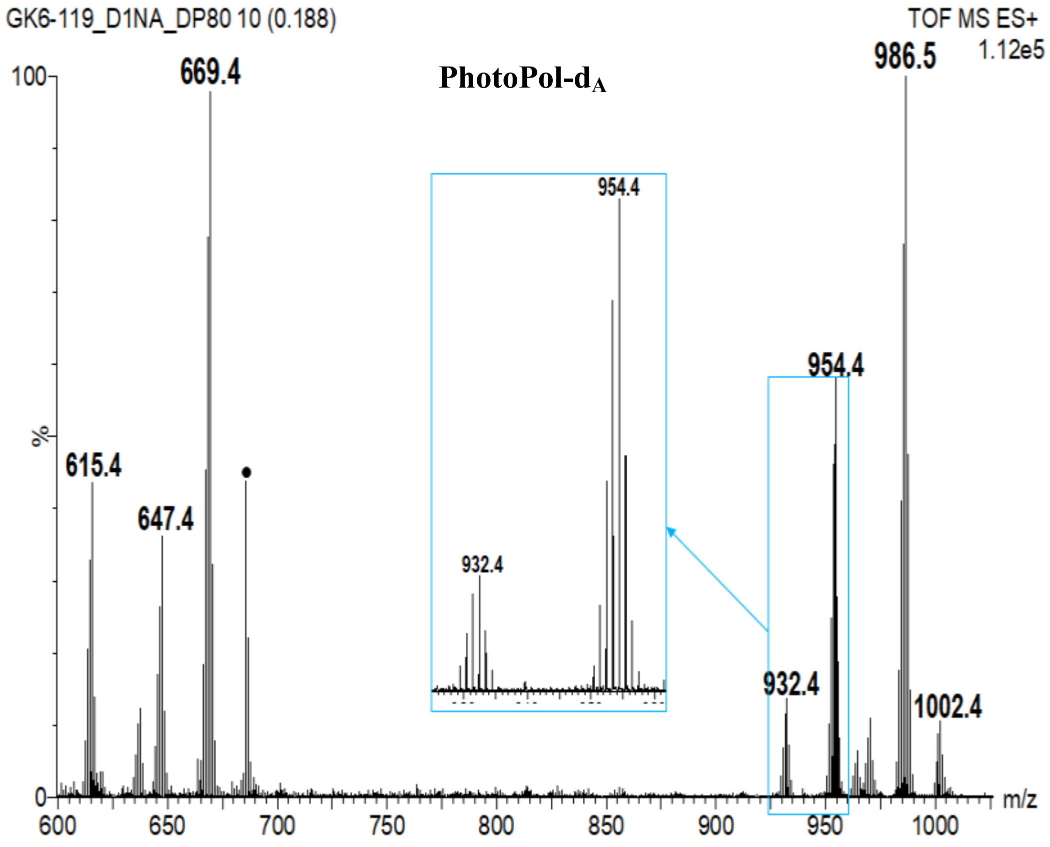

Figure S13: ESI-HRMS spectrum of **PhotoPol-d$_A$** (**12**)

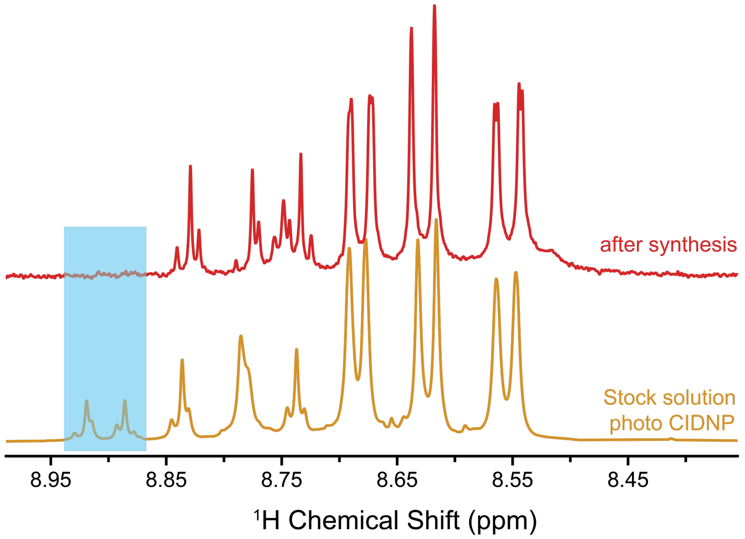

Figure S14: Comparison of ^{1}H spectra (low field region) of PhotoPol-d_A after synthesis (in $CDCl_3$) and using the stock solution used for photo-CIDNP experiments (in 1,2-tetrachloroethane-d_2). Spectra were scaled to similar intensity and aligned to clearly visualize the differences. We observe a slight degradation of the sample tentatively due to opening of the NDI ring through contact with alcohols or water. These degradation products could be the reason for the faster T_{1n} reported in Figure S2.

5) Reference:

(1) De Biasi, F.; Hope, M. A.; Avalos, C. E.; Karthikeyan, G.; Casano, G.; Mishra, A.; Badoni, S.; Stevanato, G.; Kubicki, D. J.; Milani, J.; et al. Optically Enhanced Solid-State ^{1}H NMR Spectroscopy. *J. Am. Chem. Soc.* **2023**, *145* (27), 14874-14883. DOI: 10.1021/jacs.3c03937.
(2) Yamashita, M.; Cuevas Vicario, J. V.; Hartwig, J. F. Trans Influence on the Rate of Reductive Elimination. Reductive Elimination of Amines from Isomeric Arylpalladium Amides with Unsymmetrical Coordination Spheres. *J. Am. Chem. Soc.* **2003**, *125* (52), 16347-16360. DOI: 10.1021/ja037425g.
(3) Tung, R. HETEROCYCLIC KINASE INHIBITORS. USA US 2009/0149399 A1, 2009.
